# Case report: Combined acute revascularization in early bilateral carotid stent occlusion

**DOI:** 10.3389/fneur.2022.992685

**Published:** 2022-09-16

**Authors:** David Černík, Robert Bartoš, Jarmila Neradová, Nicol Frenštátská, Filip Cihlář, Štěpánka Brušáková, Martin Sameš

**Affiliations:** ^1^Comprehensive Stroke Center, Neurology, Masaryk Hospital, Ústí nad Labem, Czechia; ^2^Department of Neurosurgery, Masaryk Hospital, J. E. Purkinje University, Ústí nad Labem, Czechia; ^3^Department of Radiology, Masaryk Hospital, J. E. Purkinje University, Ústí nad Labem, Czechia

**Keywords:** ECIC bypass, prolonged low-dose intravenous thrombolysis, carotid stent occlusion, stroke, mechanical thrombectomy

## Abstract

**Introduction:**

The introduction of a carotid stent involves the use of effective antiplatelet therapy to maintain stent patency. We present a case report of combined acute revascularization in a patient with occlusion in recently introduced stents of both carotid arteries.

**Methods:**

The patient (male, 73 years) was admitted for stroke recurrence upon discontinuation of antiplatelet therapy. According to the CTA, the closure of implanted stents of both carotid arteries was confirmed. Intravenous thrombolysis and mechanical thrombectomy were performed with complete recanalization of the left carotid stent. At 3 days apart, clinical deterioration was found with progressive stent restenosis. Percutaneous transluminal stent angioplasty, mechanical embolectomy and prolonged low-dose intravenous thrombolysis have been used repeatedly.

**Results:**

With the impossibility of maintaining the patency of carotid stents even on the maximum drug therapy and despite endovascular procedures, bilateral neurosurgical revascularization of the middle cerebral arteries using ECIC bypasses was successfully performed. Prolonged low-dose intravenous thrombolysis (20 mg recombinant plasminogen aktivator (rTPA)/10 h) has proven to be an acute bridging therapy until surgery.

**Conclusion:**

Early occlusion of the carotid stent is a significant complication of endovascular treatment of stenotic arteries. ECIC bypass revascularization of the middle cerebral artery can be a highly effective therapeutic procedure.

## Introduction

The introduction of a internal carotid artery (ICA) stent in the treatment of significant stenosis is a standard procedure. It involves the subsequent use of antiplatelet therapy to maintain stent patency. We present a case report of combined acute revascularization in a patient with occlusion in recently introduced stents of both carotid arteries.

## Methods

**Figure 1:**
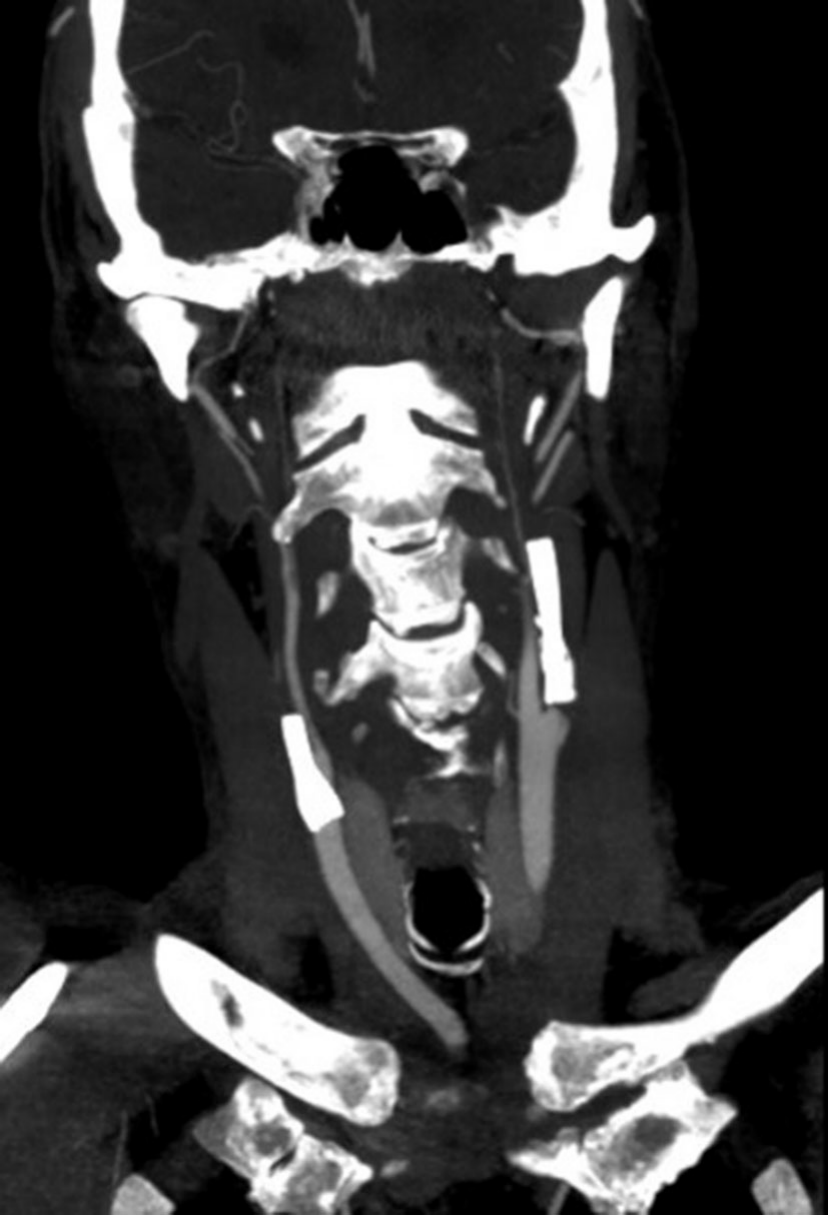
Occlusion of both carotid stents.

The case report deals with a patient (male, 73 years). The patient's medical history included arterial hypertension, ischemic heart disease, diabetes mellitus of the second type with insulin therapy, and hyperlipidemia. In August 2017, he suffered a acute ischemic stroke (AIS) during preocclusive stenosis of the right ICA. Intravenous thrombolysis (IVT, 70 mg rTPA-10 bolus and 90% by hourly infusion) was performed and acute carotid endarterectomy was performed the same day. The patient was completely free of clinical deficits the next day. In the following months, there is a gradual asymptomatic stenotization of both ICA. Endovascular treatment (stent) was considered, however, laboratory testing of the effectiveness of antiplatelet therapy repeatedly proved to be completely insufficient (acetylsalicylic acid, clopidogrel, ticlopidine, effient, prasugrel were gradually used - all were laboratory ineffective). In April 2021 he was brought with a severe AIS during occlusion of the right ICA. IVT was administered (70 mg rTPA −10 bolus and 90% by hourly infusion). The ICA was partially recanalized and ticagrelol was added to secondary prevention. The patient's clinical condition was very good (very mild left-sided hemiparesis). A stent was inserted into the ICA 8 days apart with laboratory-proven effectiveness antiplatelet therapy. One month later, due to the presumed effective antiplatelet therapy, a stent was inserted into the left asymptomatic critically stenotic ICA. At an interval of 8 days, the patient was brought in with very severe left lateralization when occlusion of both carotid stents was found (Figure 1). According to an angiographic finding, the right ICA has already been chronically occluded for many days. The etiology of a left hemisyndrome was hypoperfusion of the right hemisphere with a sudden insufficiency of collateral supply due to occlusion of the already solitary left ICA at that time. We find out at a glance that the patient has stopped the antiplatelet therapy himself. IVT was performed (70mg rTPA −10% bolus and 90% by hourly infusion) and mechanical embolectomy [MT, a large number of massive thromboemboli were repeatedly obtained by aspiration technique from the area of the stent in the left ICA and from the distal embolization in the middle cerebral artery (MCA)] was performed with complete recanalization of the left carotid stent closure, followed by continuous antithrombotic therapy with ebtifibatide. There was a significant clinical improvement in the patient.

## Results

**Figure 2:**
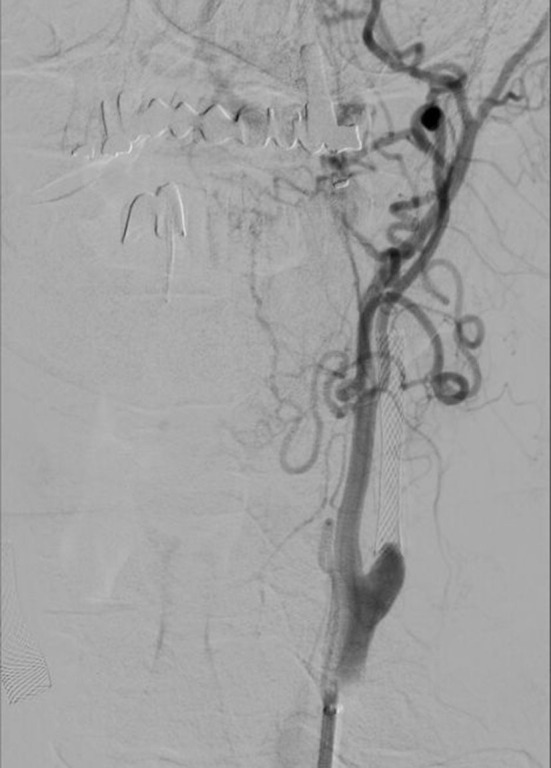
Occlusion of left carotid stent on digital subtraction angiography.

**Figure 3:**
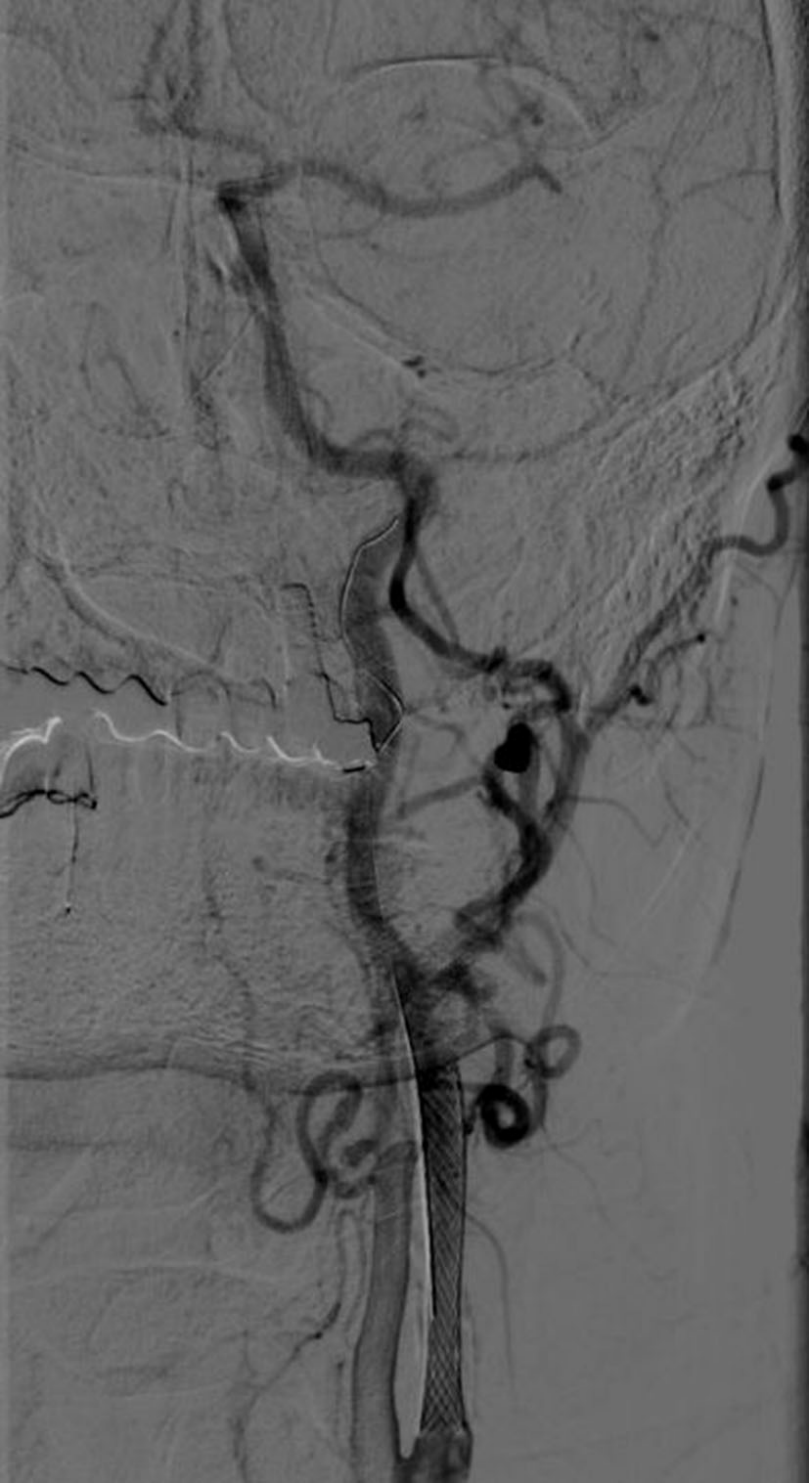
Recanalization of left carotid stent on digital subtraction angiography.

**Figure 4:**
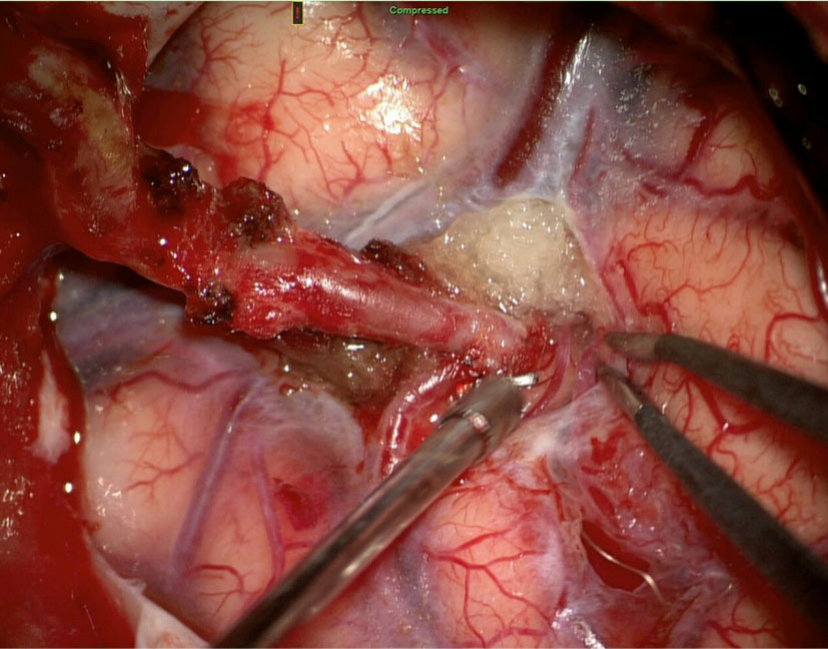
Extra-intracranial anastomosis (ECIC) of the superficialis temporalis artery—middle cerebral artery.

**Figure 5:**
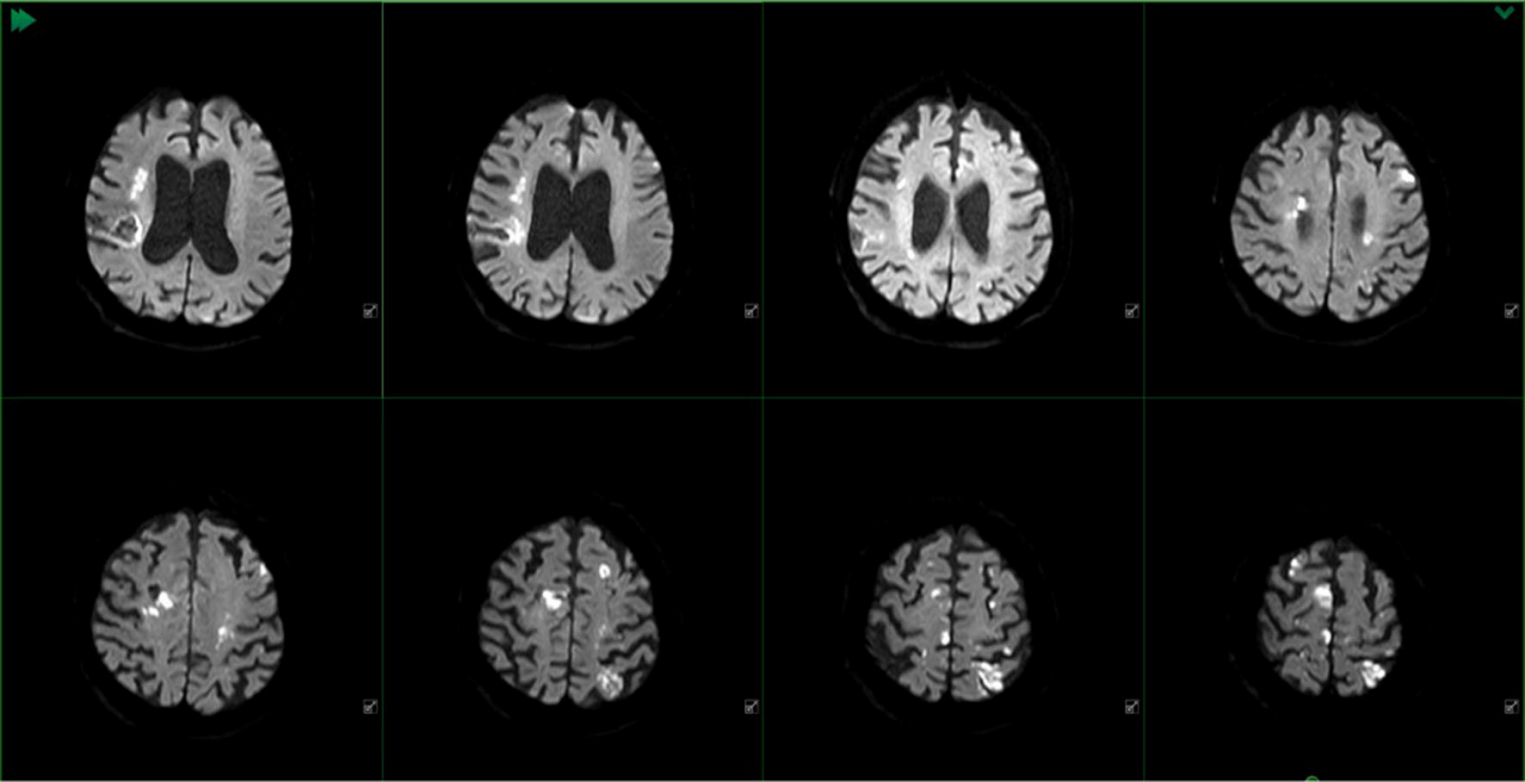
MR DWI of the patient before surgery with documentation of hemodynamic ischemia districts in both hemispheres.

At intervals of 3 days, plegia of the right limbs and aphasia suddenly develop, according to the CTA, progressive thrombosis in the stent was detected. Prolonged low-dose IVT was administered (20 mg rTPA for 10 h). Percutaneous transluminal stent angioplasty was performed simultaneously. Around 4 h after the end of IVT, the arteries are occluded, prolonged low dose IVT is administered again and the MT of the left carotid stent and middle cerebral artery is performed again (20 mg rTPA for 10 h, aspiration technique of mechanical embolectomy). Left ICA stent occlusion and its subsequent recanalization is shown in the figure (Figures 2, 3). The clinical condition was strongly dependent on blood pressure values. Transcranial Doppler ultrasonography shows signs of hypoperfusion of both cerebral hemispheres. Thus, neurosurgical revascularization—extra-intracranial anastomosis (ECIC) of the temporal artery into the middle cerebral artery basin—was performed acutely in one operation with a very good graphical result and reflection in the patient's clinical condition (Figures 4, 5). The mobility of all limbs and speech were restored and targeted rehabilitation was started for a patient with great rehabilitation potential. However, the patient subsequently refused to fully rehabilitate. Even after the intervention of the family, the cooperation does not improve and the patient died 2 months later.

## Discussion

The risk of early occlusion of the inserted carotid stent is very high. Pop et al. report early stent restenosis in 19% of cases in AIS (1). Jost et al. reported 14% reocclusions with co-infusion of eptifibatide at an early stage to prevent acute reocclusion (2). Acute stent placement is comparable to elective in ineffective antiplatelet therapy. The need for efficacy testing is presented by Collette et al. (3). The use of prolonged low-dose IVT in AIS is reported sporadically, and the results of a large randomized study are not available. The available data indicate a relatively good safety profile and efficacy of this treatment (4, 5). Acute ECIC bypass in very well-selected patients with ICA occlusion improves the chance of a good clinical outcome (6).

Among other things, our case report shows the need for patient cooperation in the rehabilitation process. The patient needs to be motivated for rehabilitation. If this is not successful despite all efforts, the hope for a good clinical result and a technically successful resolution of the brain revascularization is minimal. Our collection of patients with acute revascularization using ECIC bypass indicates a good long-term perspective for patients with a similar technical result as in this case report.

One of the reasons for the ineffectiveness of antiaggregant therapy may be the patient's non-use of medication. We should always think about this possibility, especially if resistance to several different antiplatelet drugs is detected.

In our case, there was no search for hematological abnormalities in the patient. However, with a high probability, none would have been detected—the patient admitted to not using anti-aggregation medication during hospitalization. Acute restenosis in a stent without premedication with antiaggregation therapy is known as discussed above. In the acute phase, however, even knowing the cause of thrombosis would not help us. Before stent implantation, this information would certainly be beneficial—it would disqualify the patient from elective stent implantation.

This is a case report, so a larger randomized study would be needed to draw more significant conclusions. However, the case report draws attention to the possibilities of combined therapy in a specific situation with the potential for a good clinical outcome. In our case, we can evaluate this only in the short term for the patient's death (which, however, was not caused by a recurrence of stroke).

In conclusion, early occlusion of the carotid stent is a significant complication of endovascular treatment of stenotic arteries. The use of antiplatelet therapy is careful to prevent this complication. Preventive laboratory testing of the effectiveness of antiplatelet therapy is appropriate. ECIC bypass revascularization can be a highly effective therapeutic procedure. Prolonged low-dose IVT has proven to be an acute bridging therapy until surgery. However, the absolute most important thing is to persuade the patient to follow the treatment regimen—to use regularly prescribed medication.

## Data availability statement

The raw data supporting the conclusions of this article will be made available by the authors, without undue reservation.

## Ethics statement

Ethical review and approval was not required for the study on human participants in accordance with the local legislation and institutional requirements. The patients/participants provided their written informed consent to participate in this study.

## Author contributions

DČ: manuscript writing. RB and MS: critical revision of the manuscript content (neurosurgery). JN and NF: manuscript writing, critical revision of the manuscript content and revision of english. FC: critical revision of the manuscript content (radiology). ŠB: critical revision of the manuscript content (neurology). All authors contributed to the article and approved the submitted version.

## Funding

This work was partially supported by the grant Krajská zdravotní a.s. grants IGA-KZ-2021-1-15 and IGA-KZ-2022-1-5.

## Conflict of interest

The authors declare that the research was conducted in the absence of any commercial or financial relationships that could be construed as a potential conflict of interest.

## Publisher's note

All claims expressed in this article are solely those of the authors and do not necessarily represent those of their affiliated organizations, or those of the publisher, the editors and the reviewers. Any product that may be evaluated in this article, or claim that may be made by its manufacturer, is not guaranteed or endorsed by the publisher.
